# Comparison of computed tomography features between follicular neoplasm and nodular hyperplasia

**DOI:** 10.1186/s40644-016-0089-x

**Published:** 2016-10-03

**Authors:** Kwang Hwi Lee, Dong Wook Kim, Jin Wook Baek, Yoo Jin Lee, Hye Jung Choo, Young Jun Cho, Sun Joo Lee, Young Mi Park, Soo Jin Jung, Hye Jin Baek

**Affiliations:** 1 Department of Radiology, Haeundae Paik Hospital, Inje University College of Medicine, Busan, 48108 South Korea; 2 Department of Radiology, Newoori Namsan Hospital, Busan, 48305 South Korea; 3 Department of Radiology, Busan Paik Hospital, Inje University College of Medicine, 75, Bokji-ro, Busanjin-gu, Busan, 47392 South Korea; 4 Department of Pathology, Busan Paik Hospital, Inje University College of Medicine, Busan, 48108 South Korea; 5 Department of Radiology, Gyeongsang National University Changwon Hospital, Gyeongsang National University School of Medicine, Changwon, 51476 South Korea

**Keywords:** Thyroid, Thyroid nodule, Follicular neoplasm, Nodular hyperplasia, Computed tomography

## Abstract

**Background:**

To date, appropriate management for Bethesda IV thyroid nodules is controversial, and no specific features of follicular neoplasm and nodular hyperplasia on ultrasonography, computed tomography (CT), or other imaging modalities have been reported. This study aimed to compare CT features of follicular neoplasm and nodular hyperplasia and to determine the specific CT features that could be used to distinguish follicular neoplasm from nodular hyperplasia.

**Methods:**

In 122 patients who underwent preoperative CT of the neck and thyroid surgery, 59 follicular neoplasms and 65 nodular hyperplasias were included. In each case, non-enhanced and contrast-enhanced CT images were obtained, and a single radiologist retrospectively analyzed CT images, including degree and pattern of attenuation, nodular configuration, margin, shape, pattern of calcification, degree and pattern of nodular enhancement, and CT halo sign. A univariate and multivariate logistic regression analyses were used to evaluate the predictive power of each variable and CT features with a high predictive power, respectively.

**Results:**

According to the univariate analysis, iso-attenuation, intraglandular configuration, smooth margin, ovoid shape, decreased enhancement, and absence of CT halo sign were more frequently observed in nodular hyperplasia (*p* < 0.05), whereas low attenuation, expansile configuration, lobulated margin, taller-than-wide shape, increased enhancement, and presence of computed tomography halo sign were more frequently observed in follicular neoplasm (*p* < 0.05). Multivariate analysis revealed significant differences in configuration (OR: 2.73, 1.13–6.57), degree of enhancement (OR: 2.14, 1.21–3.78), and presence of CT halo sign (OR: 7.97, 2.74–23.37) between follicular neoplasm and nodular hyperplasia (*p* < 0.05).

**Conclusions:**

Neck CT may be helpful for distinguishing follicular neoplasm from nodular hyperplasia.

**Trial registration:**

Rretrospectively registered.

## Background

Follicular neoplasm (FN) of the thyroid gland includes follicular adenoma and follicular carcinoma [1]. When FN is suspected on cytology after ultrasonography (US)-guided fine-needle aspiration (US-FNA) of a thyroid nodule (i.e., Bethesda IV class, which is characterized by a high cellularity, scant or absent colloid, and follicular cells with predominantly microfollicular or trabecular arrangements), surgery is recommended because no diagnostic method has been established to distinguish follicular carcinoma from follicular adenoma, except for surgery [2]. Herein, US-FNA has an important limitation in the preoperative detection of FN and show relatively high false-positive rate, ranging from 20 to 40 % in the literature [3–5]. The main thyroid nodule that mimics FN on cytology, is nodular hyperplasia (NH), which is characterized by a dense cellular follicular proliferation with no fibrous capsule on histopathology [6]. Furthermore, a core needle biopsy is not recommended for the management of a Bethesda IV nodule because it cannot provide useful information [7]. Therefore, unnecessary surgery for in cases of Bethesda IV nodules is not uncommon [5, 8].

Several studies have attempted to compare the imaging features between NH and FN [9–11]. Several investigators have proposed that NH can be differentiated from follicular adenoma by using US, including the ratio of solid and cystic portion, spongiform appearance, and echogenicity of thyroid nodules [9]. Other investigators have reported that ^18^F-fluorodeoxyglucose (^18^F-FDG) positron emission tomography/computed tomography (CT) can prevent a significant number of unnecessary thyroid surgeries when the nodules are larger than 2 cm or in cases of high ^18^F-FDG uptake without oncocytic changes [11]. However, no specific features of follicular neoplasm and nodular hyperplasia on US, CT, or other imaging modalities have been established. To the best of our knowledge, no comparative study of CT features between NH and FN has been published. This study aimed to compare the CT features of FN and NH and to determine the specific CT features that can distinguish FN from NH.

## Methods

### Study population

This retrospective study was approved by the Institutional Review Board, and informed consent of the patients was waived. From March 2010 to June 2014, 83 patients (male:female = 11:72, mean age: 49.7 years, age range: 28–78 years) underwent thyroid surgery in our institution for the treatment of FN. Of the 83 patients with FNs, 26 were excluded because of the absence of a preoperative neck CT scan (*n* = 11), mismatch between CT and histopathological findings (*n* = 2), small nodule size < 1 cm at the largest diameter (*n* = 8), and poor image quality of CT (*n* = 5). Finally, 59 FNs in 57 patients (male:female = 12:45, mean age: 50.4 years, range: 28–78) were included in this study.

From August 2013 to June 2014, 289 patients (male:female = 32:257, mean age: 52.2 years, age range: 22–79 years) underwent thyroid surgery for the treatment of thyroid malignancy or other thyroid lesions, and 159 had one or more NHs on histopathological analysis. Of the 159 patients, 94 were excluded because of the absence of a preoperative neck CT scan (*n* = 52), mismatch between CT and histopathological findings (*n* = 5), small nodule size < 1 cm at the largest diameter (*n* = 25), and poor image quality of CT (*n* = 12). Finally, 65 NHs in 65 patients (male:female = 5:60, mean age: 55.7 years, age range: 22–61 years) were included.

### Neck CT

Neck CT examinations were performed with multi-detector CT scanners (Discovery CT750 HD, GE Healthcare, Milwaukee, USA; Aquilion One, Toshiba Medical System Corporation, Tokyo, Japan). The scout view was scanned through the lateral projection. Both non-enhanced and contrast-enhanced images of the neck, skull base, and superior mediastinum were obtained. The acquisition parameters for Aquilion One were 120 kV; 180 mA; rotation time, 0.5 s; pitch, 0.625; field of view, 35 cm; matrix size, 512 × 512 pixel; detector collimation, 64 mm × 0.5 mm; slice thickness, 1 mm; slice increment, 1 mm. The acquisition parameters for Discovery CT750 HD were filed of view, 30 cm; slice thickness, 1.25; and slice increment, 1.25 mm. The other parameters were approximately the same as those of Aquilion One. A contrast-enhanced image was obtained after the intravenous injection of contrast media (300 mg of non-ionized iodine per milliliter, Scanlux; Sanochemia Pharmazeutika AG, Leitha, Austria) at 3 mL/s, delayed until 40 s after the initiation of contrast media.

### Image analysis

A single radiologist with 3 years of experience in head and neck imaging performed an image analysis using a picture archiving and communication system under blinded conditions for the histopathological results. The following CT features of thyroid nodules were retrospectively investigated: the degree and pattern of attenuation, configuration, margin, shape, pattern of calcification, degree and pattern of enhancement, and presence of a CT halo sign (defined as a low-attenuated rim ≥ 1 mm in thickness and surrounding ≥ 75 % of the peripheral margin of the thyroid nodule on either non-enhanced or contrast-enhanced CT image). To determine the degrees of attenuation and enhancement of thyroid nodules on non-enhanced and contrast-enhanced images, respectively, the adjacent thyroid parenchyma was used as a reference. The patterns of attenuation and enhancement of thyroid nodules on CT were classified as homogeneous or inhomogeneous. The configuration of the thyroid nodules was divided into intraglandular (the presence of thyroid parenchyma between the nodule and adjacent thyroid capsule), expansile (with a blunt angle between the lesion and adjacent thyroid capsule), or exophytic (with an acute angle between the lesion and adjacent thyroid capsule). The margin of the thyroid nodules was classified as smooth, irregular, lobulated, or poorly defined. The shape was classified based on the ratio of anteroposterior to transverse diameters in axial images as follows: (1) anteroposterior and transverse diameters of the nodule should be parallel with the anteroposterior and transverse axes of the thyroid gland, respectively. (2) the shape of the thyroid nodules was divided into ovoid (anteroposterior diameter/transverse diameter ratio ≤ 0.8), round (0.8 < anteroposterior diameter/transverse diameter ratio ≤ 1), or taller-than-wide (anteroposterior diameter/transverse diameter ratio > 1). Based on the degree of calcification, the thyroid nodule was classified as non-calcified, rim, nodular, punctate (tiny dot-like calcification), complete, or mixed (nodular plus punctate). The degree of nodular enhancement was classified as no/scant, decreased, iso-, and increased, based on a comparison with the adjacent thyroid parenchyma. CT halo sign was classified as absent or present.

To avoid investigation bias, nodule selection and image analysis were independently performed in two stages: (1) First, the selection, localization, and measurement of a thyroid nodule in all patients were performed on the basis of the CT and histopathological findings by a single radiologist. (2) After 4 weeks, image analysis of the thyroid nodules was performed by the same radiologist blinded to the histopathological findings.

### Statistical analysis

The data were tested for normal distribution by using a Kolmogorov–Smirnov test. Age at the time of diagnosis and nodular size were expressed as the mean ± standard deviation. Mean differences in age and nodule size between FN and NH were compared using the independent *t*-test. Group comparisons of categorical variables were performed using the *χ*2 test. A univariate logistic regression analysis was first used to evaluate the predictive power of each variable. CT features with a high predictive power (*p* < 0.20, Wald test) were selected and analyzed by multivariate logistic regression analysis to determine an optimal logistic regression model for distinguishing NH from FN. A receiver operating characteristic (ROC) curve analysis was performed to evaluate the diagnostic performance of CT features, and the area under the ROC curve (AUC) was compared between the two groups. All statistical analyses were performed with a statistical software (SPSS, version 17.0, SPSS, Chicago, IL, USA), and a *p* value less than 0.05 was considered statistically significant.

## Results

In the 122 patients (male:female = 14:108, mean age: 52.9 years, age range: 22–78 years) included in this study, 59 FN and 65 NH were investigated. The type of thyroid surgery included total thyroidectomy (*n* = 93), subtotal thyroidectomy (*n* = 1), and hemi-thyroidectomy (*n* = 28). The mean age of the patients with FN was significantly lower than those with NH (50.4 ± 14.9 vs 55.7 ± 11.8, *p* = 0.031). The mean nodule size was not significantly different between FN and NH (2.66 ± 1.64 cm vs 2.13 ± 1.62 cm, respectively, *p* = 0.069). Of the 59 FNs in 57 patients, 9 follicular carcinomas and 50 follicular adenomas were found. The reasons for thyroid surgery included coexisting thyroid malignancy (*n* = 9), suspicious follicular neoplasm (*n* = 25), suspicious malignancy (*n* = 8), or atypia of undetermined significance or follicular lesion of undetermined significance on cytology (*n* = 14), and patient request despite benign cytology (*n* = 1).

**Fig. 1:**
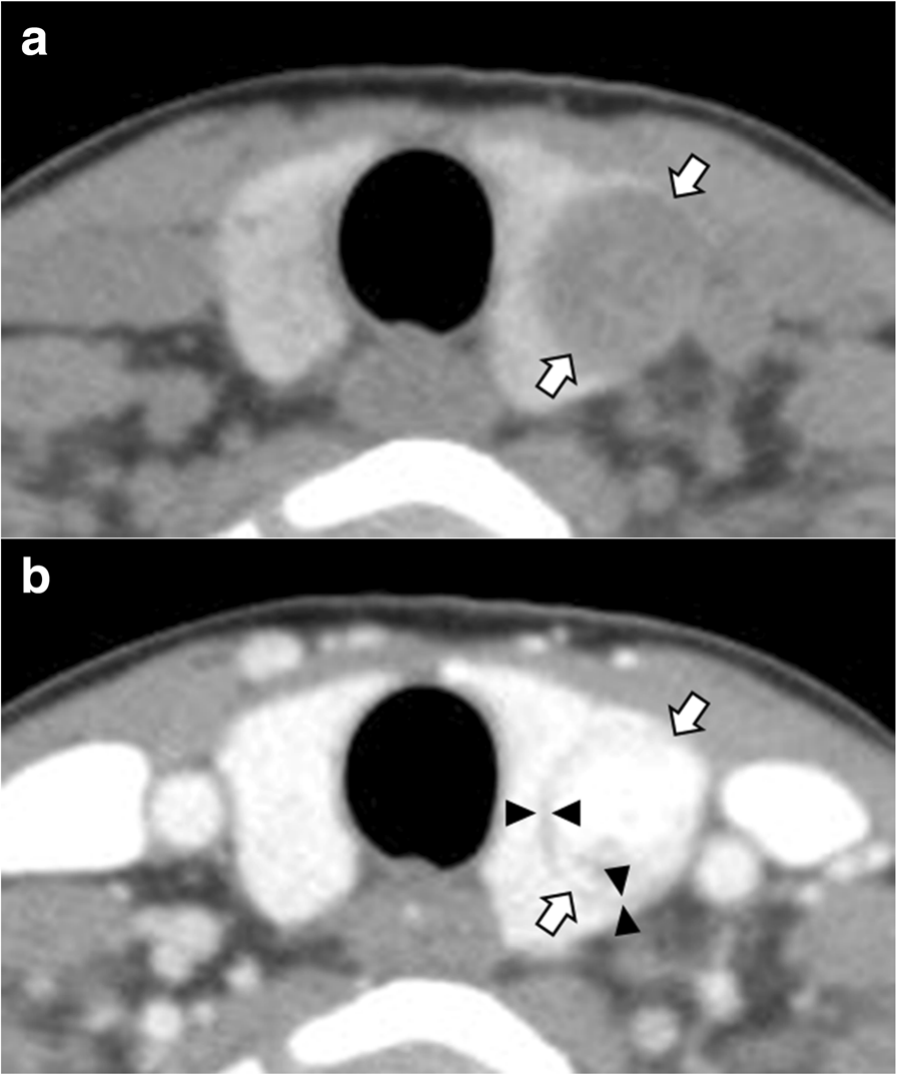
A 51-year-old woman with follicular adenoma in the left thyroid lobe (largest diameter, 1.9 cm). In non-enhanced (**a**) and contrast-enhanced (**b**) axial CT images, follicular adenoma (arrows) in the left thyroid lobe shows homogeneous low attenuation, mildly expansile configuration, taller-than-wide shape, inhomogeneous and increased enhancement, and presence of the CT halo sign (arrowheads)

**Fig. 2:**
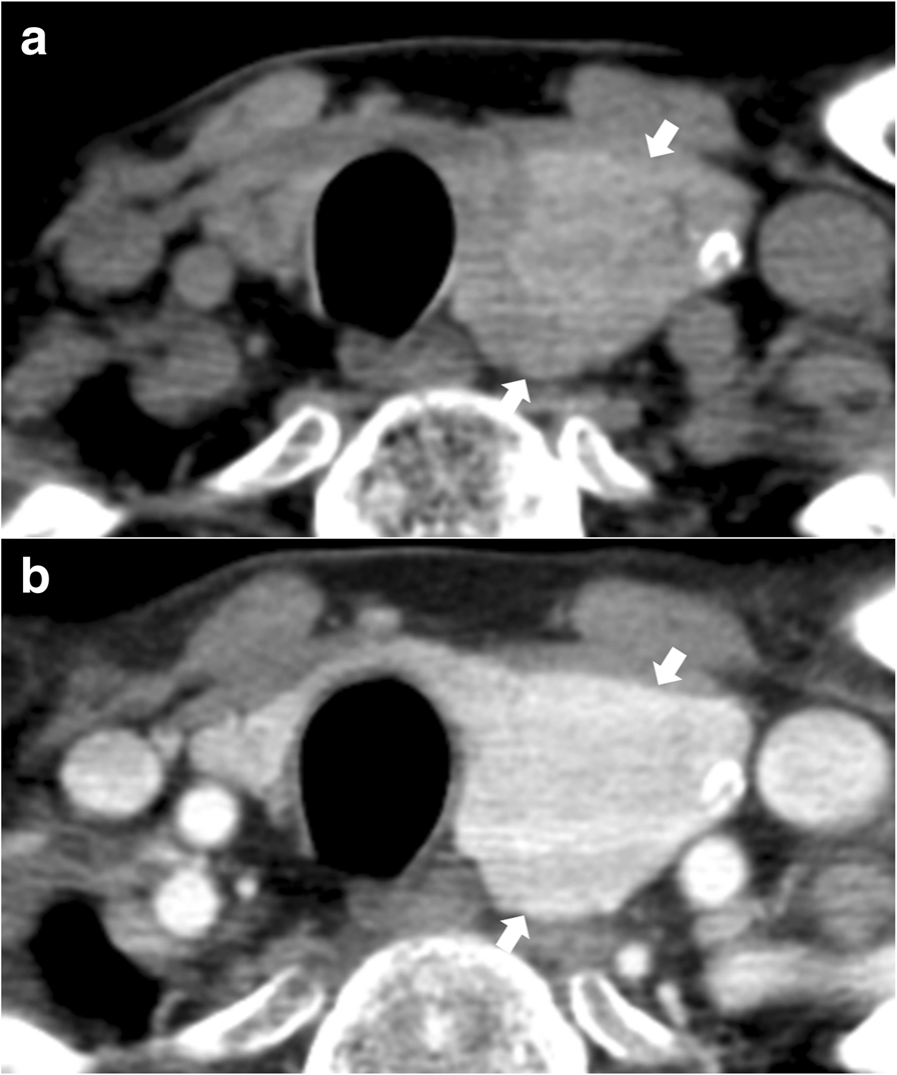
A 62-year-old man with nodular hyperplasia in the left thyroid lobe (largest diameter, 3.5 cm). In non-enhanced (**a**) and contrast-enhanced (**b**) axial CT images, nodular hyperplasia (arrows) in the left thyroid lobe shows inhomogeneous iso-attenuation, ovoid shape, homogeneous iso-enhancement, and absence of the CT halo sign

The CT features of FN and NH are listed in Table 1. CT features common to both FN and NH included low and inhomogeneous attenuation, smooth margin, round shape, no calcification, and inhomogeneous enhancement. The prevalence of expansile configuration, increased enhancement, and presence of the CT halo sign was higher in FN (Fig. 1), whereas the prevalence of intraglandular configuration, decreased enhancement, and absence of the CT halo sign was higher in NH (Fig. 2). In particular, the CT halo sign was more frequently observed in FN than NH. In univariate analysis, degree of attenuation, configuration, margin, shape, degree of enhancement, and the CT halo sign were significantly different between FN and NH (*p* < 0.05, Table 1). Iso-attenuation, intraglandular configuration, smooth margin, ovoid shape, decreased enhancement, and absence of the CT halo sign were more frequent in NH, whereas low attenuation, expansile configuration, lobulated margin, taller-than-wide shape, increased enhancement, and presence of the CT halo sign were more frequent in FN. However, pattern of attenuation, calcification, and pattern of enhancement were not significantly different between FN and NH (*p* > 0.05). In multivariate analysis, configuration (OR: 2.73, 1.13–6.57, *p* = 0.026), degree of enhancement (OR: 2.14, 1.21–3.78, *p* = 0.009), and the CT halo sign (OR: 7.97, 2.74–23.37, *p* < 0.001) were significantly different between FN and NH (Table 2).

**Table 1:** Univariate logistic regression analysis of computed tomography features of nodular hyperplasia and follicular neoplasm

CT features	Nodular hyperplasia (*n* = 65)	Follicular neoplasm (*n* = 59)	*p* value
Degree of attenuation			
no visualization	5 (7.7 %)	0 (0 %)	<0.001
low	44 (67.7 %)	57 (96.6 %)	
iso-	15 (23.1 %)	1 (1.7 %)	
high	1 (1.5 %)	1 (1.7 %)	
Pattern of attenuation			
homogeneous	25 (38.4 %)	19 (32.2 %)	0.466
inhomogeneous	40 (61.6 %)	40 (67.8 %)	
Configuration			
intraglandular	41 (63.1 %)	21 (35.6 %)	0.003
expansile	18 (27.7 %)	34 (57.6 %)	
exophytic	6 (9.2 %)	4 (6.8 %)	
Margin			
smooth	42 (64.7 %)	34 (57.6 %)	0.023
irregular	1 (1.5 %)	0 (0 %)	
lobulated	16 (24.6 %)	25 (42.4 %)	
poorly defined	6 (9.2 %)	0 (0 %)	
Shape			
ovoid	26 (40 %)	10 (16.9 %)	0.008
round	29 (44.6 %)	30 (50.9 %)	
taller-than-wide	10 (15.4 %)	19 (32.2 %)	
Calcifications			
none	59 (90.8 %)	48 (81.3 %)	0.186
rim (eggshell)	1 (1.5 %)	2 (3.4 %)	
nodular	2 (3.1 %)	6 (10.2 %)	
punctate	2 (3.1 %)	0 (0 %)	
mixed^*^	1 (1.5 %)	3 (5.1 %)	
Degree of enhancement			
no/scant	4 (6.2 %)	0 (0 %)	< 0.001
decreased	29 (44.5 %)	18 (30.5 %)	
iso-	25 (38.5 %)	15 (25.4 %)	
increased	7 (10.8 %)	26 (44.1 %)	
Pattern of enhancement			
homogeneous	13 (20 %)	4 (6.8 %)	0.06
inhomogeneous	52 (80 %)	55 (93.2 %)	
CT halo sign			
absent	54 (83.1 %)	17 (28.8 %)	< 0.001
present	11 (16.9 %)	42 (71.2 %)	

*Note*. — ^*^, ‘Mixed’ means punctate plus nodular calcifications. Data are number of items, with percentage in parentheses. *CT* computed tomography

**Table 2:** Multivariate logistic regression analysis of computed tomography features for distinguishing follicular neoplasm from nodular hyperplasia

CT features	Odds Ratio*	*p* value
Type of attenuation	0.40 (0.11, 1.40)	0.149
Pattern of attenuation	0.58 (0.20, 1.72)	0.328
Configuration	2.73 (1.13, 6.57)	0.026
Margin	0.72 (0.43,1.22)	0.224
Shape	1.88 (0.89, 3.98)	0.098
Calcifications	1.44 (0.90. 2.23)	0.128
Degree of enhancement	2.14 (1.21, 3.78)	0.009
Pattern of enhancement	1.95 (0.422, 3.02)	0.393
CT halo sign	7.97 (2.74, 23.37)	< 0.001

*Note*. — *, Numbers in parentheses are 95 % confidence intervals. *CT* computed tomography

The diagnostic indices of individual CT features for distinguishing FN from NH are presented in Table 3. The AUC values for expansile configuration, taller-than-wide shape, increased enhancement, and presence of the CT halo sign were significantly higher in FN (95 % CI, *p* < 0.05).

**Table 3:** Diagnostic performance of computed tomography features for distinguishing follicular neoplasm from nodular hyperplasia

CT features	Sensitivity (%)	Specificity (%)	PPV (%)	NPV (%)	*A*_*z*_ value*	*p* value
Degree of attenuation	96.6	24.6	53.8	88.9	0.567	0.058
Pattern of attenuation	67.8	38.5	50	56.8	0.531	0.469
Configuration	64.4	63.1	61.3	66.1	0.620	0.009
Margin	0	90.8	0	50	0.519	0.679
Shape	83.1	40	55.7	72.2	0.68	0.001
Calcifications	18.6	90.8	64.7	55.1	0.546	0.142
Degree of enhancement	44.1	89.2	78.8	63.7	0.682	< 0.001
Pattern of enhancement	93.2	20	51.4	76.5	0.559	0.058
Presence of CT halo sign	71.2	83.1	79.2	76.1	0.771	< 0.001

*Note*. — *, *A*_*z*_ means the largest area under the receiver operating characteristic curve. *CT* computed tomography, *PPV* positive predictive value, *NPV* negative predictive value

## Discussion

US-FNA has been established as the first method of choice for evaluating sonographically suspicious thyroid nodules. For reporting thyroid cytopathology, the Bethesda system, including 6 classes, has been used worldwide [2]. Although the Bethesda system has discrete advantage in terms of communication between cytopathologists, physicians, and other clinicians, several limitations should be considered. One of the limitations is that Bethesda IV nodules include a significant proportion of non-neoplastic lesions (up to 35 %) [2, 5, 12]. NH is often classified as Bethesda class IV, because it has a similar histopathological appearance to FN, and the absence of a fibrous capsule cannot be evaluated on cytology [6, 13]. In this case, core needle biopsy is not helpful for identifying NH among Bethesda class IV nodules, resulting in unnecessary thyroid surgery [7].

Several investigators have attempted to distinguish malignant thyroid nodules from benign nodules on CT [14–16]. They suggested that calcification, taller-than-wide shape, and high degree of nodular enhancement were meaningful CT features for thyroid malignancy [15, 16]. Although previous CT studies have focused on distinguishing malignancy from benign nodules, the focus of our study was to determine specific CT features for distinguishing FN from a non-neoplastic nodule. The results of this study showed that the combination of expansile configuration, increased enhancement, and the presence of a CT halo sign suggests the possibility of FN rather than NH. In terms of the configuration and shape of thyroid nodules, FN tends to reveal expansile configuration and taller-than-wide shape, whereas NH tends to represent intraglandular configuration and ovoid shape. This difference may originate from the essentials of NH and FN: FN is a neoplasm of the thyroid gland, whereas NH is a non-neoplastic condition [17, 18]. Regarding CT enhancement of thyroid nodules, increased enhancement was more frequently observed in FN than in NH. This finding is concordant with that of a previous study, in which FN tended to have less cystic degeneration and scant colloid in comparison with NH [19].

The well-known US halo sign (i.e., hypoechoic rim around thyroid nodules) is generated by a pseudocapsule of fibrous connective tissue, which appears as compressed normal adjunct parenchyma or inflammatory cell infiltration on histopathology [20, 21]. One study reported that a complete, even halo sign was suggestive of benignity with specificity of 95 % [22], whereas another study reported that more than half of benign nodular thyroid diseases revealed no US halo sign [21]. In the present study, we first used the CT halo sign of a thyroid nodule. The CT halo sign was observed in both FN and NH, but their frequency was higher in FN (71.2 %, 42/59). The prevalence rate of the halo sign in FN was higher than that of the US halo sign (50 %, 21/42) in a previous study [9]. In our study, the prevalence rate of the CT halo sign was higher in FN (71.2 %, 42/59) than in NH (16.9 %, 11/54). This might be because FN has a high incidence of discrete fibrous capsule, whereas NH has a low incidence of discrete fibrous capsule. For clarity, further studies, including a larger number of cases, should be required. Separately, further CT studies using multiple phase or dynamic enhancement may be necessary for evaluating specific enhancing pattern of NH or FN.

This study has several limitations. First, the analysis of CT features was performed by a single radiologist. For clarity, a multicenter study should be conducted in the future. Second, all patients underwent thyroid surgery. Furthermore, thyroid nodules < 1 cm at the largest diameter were not included. Thus, selection bias could not be avoided. Third, the analysis of attenuation and enhancement in thyroid nodules was performed only by visual assessment. Hounsfield unit of a thyroid nodule in the evaluation of attenuation or enhancement was not measured. Finally, the presence or absence of underlying diffuse thyroid disease was not considered. Because the adjacent thyroid parenchyma was used as a reference to analyze the degree of attenuation and enhancement, visual assessment of nodules might be affected according to the presence of an underlying diffuse thyroid disease.

## Conclusions

The results of this study showed that FN is indicated by expansile configuration, increased enhancement, and presence of the CT halo sign, whereas NH is indicated by intraglandular configuration, decreased enhancement, and absence of the CT halo sign. Therefore, neck CT may be helpful to differentiate FN from NH.

## Abbreviations

CT: Computed tomography
FN: Follicular neoplasm
FNA: Fine-needle aspiration
NH: Nodular hyperplasia
US: Ultrasonography

## References

[1] Thompson LD, Wieneke JA, Paal E, Frommelt RA, Adair CF, Heffess CS (2001). A clinicopathologic study of minimally invasive follicular carcinoma of the thyroid gland with a review of the english literature. Cancer.

[2] Cibas ES, Ali SZ, Conference NCITFSotS (2009). The Bethesda system for reporting thyroid cytopathology. Am J Clin Pathol.

[3] Deveci MS, Deveci G, LiVolsi VA, Baloch ZW (2006). Fine-needle aspiration of follicular lesions of the thyroid: diagnosis and follow-Up. Cytojournal.

[4] Dabelic N, Matesa N, Matesa-Anic D, Kusic Z (2010). Malignancy risk assessment in adenomatoid nodules and suspicious follicular lesions of the thyroid obtained by fine needle aspiration cytology. Coll Antropol.

[5] Yoon RG, Baek JH, Lee JH, Choi YJ, Hong MJ, Song DE (2014). Diagnosis of thyroid follicular neoplasm: fine-needle aspiration versus core-needle biopsy. Thyroid.

[6] Schreiner AM, Yang GC (2012). Adenomatoid nodules are the main cause for discrepant histology in 234 thyroid fine-needle aspirates reported as follicular neoplasm. Diagn Cytopathol.

[7] Min HS, Kim JH, Ryoo I, Jung SL, Jung CK (2014). The role of core needle biopsy in the preoperative diagnosis of follicular neoplasm of the thyroid. APMIS.

[8] Sahin M, Gursoy A, Tutuncu NB, Guvener DN (2006). Prevalence and prediction of malignancy in cytologically indeterminate thyroid nodules. Clin Endocrinol (Oxf).

[9] Moon WJ, Kwag HJ, Na DG (2009). Are there any specific ultrasound findings of nodular hyperplasia (“leave me alone” lesion) to differentiate it from follicular adenoma?. Acta Radiol.

[10] Seo HS, Lee DH, Park SH, Min HS, Na DG (2009). Thyroid follicular neoplasm: can sonography distinguish between adenomas and carcinomas?. J Clin Ultrasound.

[11] Munoz Perez N, Villar del Moral JM, Muros Fuentes MA, Lopez de la Torre M, Arcelus Martinez JI, Becerra Massare P (2013). Could 18F-FDG-PET/CT avoid unnecessary thyroidectomies in patients with cytological diagnosis of follicular neoplasm?. Langenbecks Arch Surg.

[12] Yang GC, Liebeskind D, Messina AV (2003). Should cytopathologists stop reporting follicular neoplasms on fine-needle aspiration of the thyroid?. Cancer.

[13] Suster S (2006). Thyroid tumors with a follicular growth pattern: problems in differential diagnosis. Arch Pathol Lab Med.

[14] Shetty SK, Maher MM, Hahn PF, Halpern EF, Aquino SL (2006). Significance of incidental thyroid lesions detected on CT: correlation among CT, sonography, and pathology. AJR Am J Roentgenol.

[15] Yoon DY, Chang SK, Choi CS, Yun EJ, Seo YL, Nam ES (2008). The prevalence and significance of incidental thyroid nodules identified on computed tomography. J Comput Assist Tomogr.

[16] Kim DW, Jung SJ, Baek HJ (2015). Computed tomography features of benign and malignant solid thyroid nodules. Acta Radiol.

[17] Kim EK, Park CS, Chung WY, Oh KK, Kim DI, Lee JT (2002). New sonographic criteria for recommending fine-needle aspiration biopsy of nonpalpable solid nodules of the thyroid. AJR Am J Roentgenol.

[18] Alexander EK, Marqusee E, Orcutt J, Benson CB, Frates MC, Doubilet PM (2004). Thyroid nodule shape and prediction of malignancy. Thyroid.

[19] Wu S, DeMay RM, Papas P, Yan B, Reeves W (2012). Follicular lesions of the thyroid: a retrospective study of 1,348 fine needle aspiration biopsies. Diagn Cytopathol.

[20] Propper RA, Skolnick ML, Weinstein BJ, Dekker A (1980). The nonspecificity of the thyroid halo sign. J Clin Ultrasound.

[21] Hoang JK, Lee WK, Lee M, Johnson D, Farrell S (2007). US Features of thyroid malignancy: pearls and pitfalls. Radiographics.

[22] Lu C, Chang TC, Hsiao YL, Kuo MS (1994). Ultrasonographic findings of papillary thyroid carcinoma and their relation to pathologic changes. J Formos Med Assoc.

